# Estrogenic Pastures: A Source of Endocrine Disruption in Sheep Reproduction

**DOI:** 10.3389/fendo.2022.880861

**Published:** 2022-04-28

**Authors:** Kelsey R. Pool, Faustine Chazal, Jeremy T. Smith, Dominique Blache

**Affiliations:** School of Agriculture and Environment M087, University of Western Australia Institute of Agriculture, The University of Western Australia, Crawley, WA, Australia

**Keywords:** phytoestrogen, reproduction, endocrine disruptor, clover disease, ruminant

## Abstract

Phytoestrogens can impact on reproductive health due to their structural similarity to estradiol. Initially identified in sheep consuming estrogenic pasture, phytoestrogens are known to influence reproductive capacity in numerous species. Estrogenic pastures continue to persist in sheep production systems, yet there has been little headway in our understanding of the underlying mechanisms that link phytoestrogens with compromised reproduction in sheep. Here we review the known and postulated actions of phytoestrogens on reproduction, with particular focus on competitive binding with nuclear and non-nuclear estrogen receptors, modifications to the epigenome, and the downstream impacts on normal physiological function. The review examines the evidence that phytoestrogens cause reproductive dysfunction in both the sexes, and that outcomes depend on the developmental period when an individual is exposed to phytoestrogen.

## Introduction

The endocrine disrupting effects of phytoestrogens has been of emerging interest in both animal production and human health. The full spectrum of effects on reproduction continue to be elucidated. Initially of interest in human reproduction, the impact of phytoestrogens on reproduction is becoming increasingly relevant in livestock production because several key pasture species are estrogenic. The effects of estrogenic pasture on livestock reproduction are a considerable economic and animal welfare issue.

Several species of subterranean clover (“sub clovers”) were incorporated into Australian livestock grazing systems in the 19^th^ century (1, 2). These sub clovers were widely adopted due to their nutritive value as pasture legumes, their resilient characteristics such as the ability to remain dormant over many seasons, their lower requirement for fertilizer than other pasture species, and their persistence under grazing (1). As a result, sub clover pastures are well integrated into animal production systems in Australia. Many of those species are now recognized to have high levels of the isoflavone formononetin, which is metabolized to equol in the rumen. Equol causes a spectrum of moderate to severe functional and morphological changes to the ewe reproductive tract (3–5) that has been termed “clover disease”. Although it is suspected that clover disease continues to cause fertility and welfare issues in sheep, investigation into it has been dormant since the 1990’s.

The female has been the focus of livestock research into phytoestrogen effects on reproduction. Early investigations into the disruptive effects of estrogenic clover described masculinized genitalia, morphological changes to the cervix, and irregularities in the duration of the estrous cycle (3, 4, 6, 7). While there have been reports on the profound effects of phytoestrogens on male fertility in several species (8–10) and *in vitro* (11), there has been very little investigation into reproductive compromise in the ram.

The obvious solution to mitigate clover disease in sheep production is pasture renovation to eliminate the sub clover cultivars that are estrogenic. However, pasture renovation can take several years, and does not guarantee that estrogenic sub clover will not persist or re-emerge, given the resilient characteristics of this species (1).

Though the known presentation of clover disease has changed over time, with reduced severity compared to initial reports (4, 12, 13), an impact on sheep reproduction is likely still present (14). With the expansion of research into the impacts of other endocrine disruptors on reproduction (15, 16), new perspectives emerge on the potential mechanisms behind how estrogenic pasture influences on sheep reproduction. The first step toward prevention of the issue, therefore, is to define the severity, nature, and distribution of clover disease in commercial systems, to establish the mechanistic basis behind the phenomenon, and to increase awareness of the potential impacts of estrogenic sub clover within the industry. Accordingly, this review examines phytoestrogens as endocrine disruptors in sheep reproduction, with reference to known and postulated mechanisms of phytoestrogens in both the sexes.

## Ovine Clover Disease: The First Evidence for Phytoestrogens as Endocrine Disruptors in Sheep

**Figure 1:**
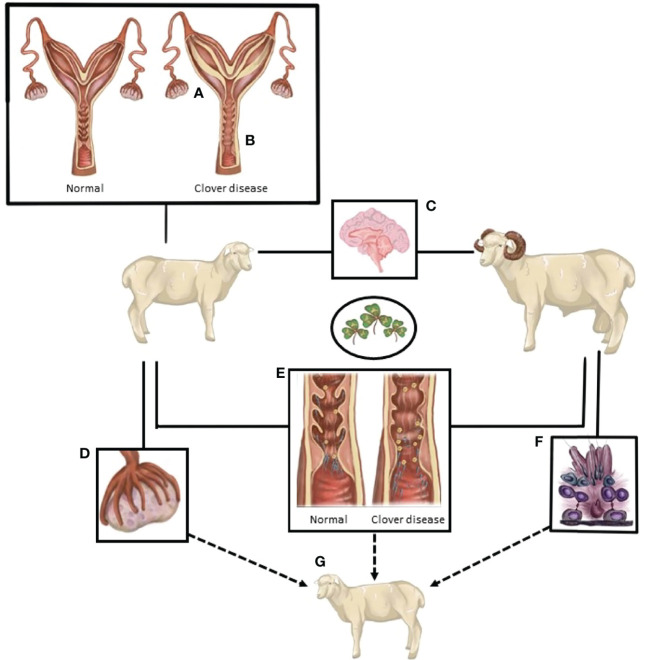
A summary of the known and postulated sites of action of compounds from oestrogenic pasture that lead to compromised sheep reproduction. **(A)** Endometrial thickening/edema. **(B)** Loss of cervical folds. **(C)** Excessive oestrogen-like actions in the neuroendocrine control of reproduction. **(D)** Follicle development, quality and potentially ovulatory ability are reduced. **(E)** The interaction between spermatozoa and the female tract is altered. Loss of cervical crypts, changes in mucus composition, and consistency and changes in the female immune response hinder sperm navigation of the female tract. **(F)** Sperm production and quality is potentially reduced. **(G)** Exposure of both male and female gametes, and the ovine embryo, may cause differential developmental programming of the subsequent generation.

Ovine clover disease was first detected and described in Western Australia in the 1940’s (17). Decades later, the disease was attributed to the high formononetin content of several cultivars of *Trifolium subterraneum*, a common pasture legume in sheep production systems in Australia (3, 4, 6, 7, 18). In the rumen, formononetin is converted to the estrogenic metabolite equol, causing masculinized genitalia, morphological changes to the cervix (**Figures 1A, B**), and irregularities in the duration of the estrous cycle (3, 4, 6, 7). These changes are accompanied by a reduction in the fertility rate, an increase in dystocia, an increase in the number of stillborn lambs, and an increase in the incidence of uterine prolapse after parturition (3, 17–19). Though these studies were critical in identifying the role of estrogenic sub clover in the compromise of sheep reproduction, the mechanisms behind the disease are not fully understood. Though the severe manifestations of clover disease appear to have been eradicated, anecdotal reports indicate that more subtle manifestations of sub-fertility have persisted when sheep graze on sub clover cultivars that are estrogenic.

Despite evidence that clover disease remains an issue in Australian sheep production systems (14), there has been little progress toward understanding the disease or developing solutions that will mitigate the impact on ovine fertility. Investigations in other species have revealed that phytoestrogens operate *via* diverse pathways, including competitive binding to nuclear estrogen receptors, that can result in both agonistic and antagonistic actions, as well as by modifying the epigenome. While these studies in other species confirm that exposure to phytoestrogens has detrimental effects on reproduction, particularly during some sensitive developmental periods, there remains a lack of comprehensive evidence on the causal pathways.

## A Brief Overview of Phytoestrogens and their Predicted Modes of Action

New additions continue to be made regularly to the spectrum of plant-derived estrogenic compounds that are known broadly as phytoestrogens. The predominant classes of phytoestrogens are isoflavones (daidzein, formononetin, genistein, glycitein), flavones (luteolin), flavonoids (quercetin, kaempferol), coumestans (coumestrol), stilbenes (resveratrol), and lignans (lariciresinol, matairesinol, pinoresinol, secoisolariciresinol) (20, 21). When they are consumed by an animal, many of these compounds can be further metabolized by the gut microflora into estrogenic metabolites. An example of particular relevance is the estrogenic by-product equol, resulting from the breakdown of formononetin in the mammalian digestive tract (22, 23).

The mechanism(s) that link phytoestrogens and reproduction remain controversial, though several key pathways have been proposed *via* which estrogenic compounds could operate. Perhaps the most discussed pathway is the interaction of phytoestrogens with estrogen receptors, albeit with a far lower binding affinity than the endogenous ligand, 17‐β‐estradiol (24, 25). In vertebrates, there are two main subtypes of nuclear estrogen receptor, ERα and ERβ (which are comprehensively reviewed by 26). The biological functions of the two receptor subtypes vary, as does their distribution between tissues, between the sexes, and between species (27–29). When they are activated by a ligand, the nuclear receptors ERα and ERβ are thought to act through either direct genomic modulation or indirect signaling. In the former, when ligand binds to the estrogen-receptor, the receptor dimerizes and binds to estrogen response elements in the promoter region of specific genes, thereby stimulating or repressing the transcription of that gene (30, 31). Indirect signaling leads to changes in transcription without a direct interaction with DNA (32, 33). Importantly, phytoestrogens not only competitively bind with ERα and ERβ, but can promote transcription from this interaction (34, 35).

Many of estrogens actions occur after stimulation of ERα. Stimulation of ERα is known to induce cellular proliferation, including the priming of the uterus *via* uterine cell production (36) and cancer progression, particularly in breast tissue, reproductive tissues, and bone (37–39). Estrogen- ERα binding is also able to induce estrogen-neuroendocrine feedback in the hypothalamus (40, 41), and increase transcription in most cell types (42). While ERβ is often co-expressed with ERα (26, 43), the stimulation of ERβ is thought to oppose the actions of ERα on gene expression (26, 31, 44–46). Thus, the outcome of estrogen stimulation for a particular cell will depend on the relative expression of ERα and ERβ. Both subtypes are involved in glucose (47–49) and lipid (50, 51) homeostasis in the brain, liver, pancreas, heart, and skeletal muscle (52). Most phytoestrogens have a higher binding affinity for ERβ than ERα (53), though that bias does not clearly translate to predictable physiological outcomes *in vivo* in mammalian systems. Notably, the expression of both receptor subtypes is increased by increased estrogen exposure (26), which may be a crucial mechanism behind the cumulative pathology resulting from long-term phytoestrogen exposure (3, 6). In the context of estrogenic pasture, an increase in receptor expression could underly the clinical manifestations, whereby long-term phytoestrogen exposure confers permanent infertility in the ewe, rather than the transient infertility that is observed with short-term exposure.

In addition to actions mediated by ERα and ERβ, phytoestrogens can interact with the G protein-coupled estrogen receptor (GPER). While ERα and ERβ are nuclear receptors, and therefore require their ligands to access the nucleus, GPER is a membrane associated receptor that is distributed across a multitude of tissues, at least in humans and rodents (54–57). When it binds ligand, GPER operates through non-genomic signaling, a comprehensive discussion of which can be found in a recent review by Luo and Liu (54). Of note, GPER is present in the reproductive tissues of both sexes, including breast tissue (58–60), the testis (61), prostate (62), ovary (63, 64), and endometrium (65, 66). Exogenous estrogenic compounds, including genistein, bisphenol A, and zearalenone, have a similar binding affinity for GPER as they do for the nuclear estrogen receptors (67). Of interest, some phytoestrogens, including equol and genistein, have been reported to have a greater binding affinity for GPER compared to 17β*-*estradiol (68). Thus, these extra-nuclear estrogen receptors may be a significant pathway through which exogenous estrogens can exert endocrine-disruptive effects through non-genomic, or indirect transcriptional pathways.

Phytoestrogens could also alter reproductive function *via* an indirect effect on genome transcription (69) that is often referred to as organizational effects. Modifications to the epigenome, such as DNA methylation, non-coding RNA’s, and histone modification, can influence the expression of the genome without altering the DNA sequence (70, 71). There is evidence that endocrine disrupting agents, and specifically exogenous estrogenic compounds, alter the epigenome in several tissues. For example, in neonatal rodents, coumestrol, genistein, and the estrogenic metabolite equol induce hypermethylation in several regions of the genome, including tissue-specific alterations in the uterus, kidney, and pancreas (72–76), as well as broadly increasing DNA methylation in the epigenome (73), in proto-oncogenes (72), and in dermal tissue (74). In addition to the endocrine disrupting actions of phytoestrogens, it is likely that *in utero* exposure to estrogenic compounds is detrimental to reproductive outcomes, given that gestation is an important period of epigenetic remodeling. Phytoestrogens can cross the blood-placenta barrier and infiltrate fetal tissues, including the brain (77, 78). To date, the phenotypic consequences of the epigenetic effects of phytoestrogen exposure have not been investigated.

It is therefore possible that epigenetic modifications that follow phytoestrogen exposure will have biologically relevant effects on the ovine reproductive system. Epigenetic changes that are induced by environmental factors, such as by assisted reproductive technologies and climate, are known to modify sperm function (79, 80), reproductive success in both male and female (81, 82), and disease onset (83) across a range of mammalian species. Moreover, these modifications can persist through generations (83, 84). Targeted and broad epigenetic modifications can influence the clinical severity of other reproductive pathologies, such as varicocele (85), polycystic ovary syndrome (86), and endometriosis (87). Theoretically, epigenetic changes in the reproductive system of sheep after exposure to phyto-estrogens could be at least partially responsible for the wide variation in clinical manifestations of clover disease, and could account for the persistence of flock sub-fertility even after sheep are removed from clover pasture.

Thus, the effects of phytoestrogens can be exerted *via* several mechanisms and pathways. An understanding of the systemic and direct mechanisms that lead from phytoestrogen exposure to reproductive pathology will be essential to find solutions. The developmental period during which an individual is exposed to phytoestrogens is likely critical in the extent of reproductive dysfunction. Developmentally sensitive periods include uterine life, neonatal life, prior to and during puberty, and times when estrogen has an essential biological role, such as during the estrous cycle.

## The Timing of Phytoestrogen Exposure Is Critical for Reproductive Outcomes

### Maternal Exposure to Phytoestrogens During Pregnancy, in the Neonate, and Prior to the Onset of Puberty

Beneficial and detrimental outcomes on female fertility after exposure to phytoestrogens have been reported. That apparent contradiction is probably due to the timing, duration, and level of phytoestrogen exposure, with consequences dependent on whether the individual is exposed *in utero*, during the neonatal and prepubertal periods, or during adult life.

The widespread use of soy-based infant formula, which is now known to contain physiologically relevant concentrations of the isoflavones genistein and genistin, has stimulated research on phytoestrogen exposure during key developmental periods in humans, and to a much larger extent in rodents (88, 89). Infants that consume soy-based formula have circulating levels of genistein that are 13,000 to 68,000-fold higher than the normal biological concentration of estradiol in non-exposed infants in the same age bracket (88). While the long term effects of phytoestrogen exposure in human infants are not known, *in utero* and neonatal exposure to genistein in rodents induces morphological alterations in the reproductive tract, including precocious vaginal opening (90), ovarian follicle atresia (91), increased uterine fluid content (92), and hyperplasia of the endometrium (92–94).

Given those outcomes in humans and rodents, it is plausible that phytoestrogen exposure during the pre-pubertal period could alter the onset of puberty in the ewe-lamb. The onset of puberty in the ewe requires considerable modulation of the hypothalamic-hypophyseal-gonadal axis and is subject to complex interactions between endocrine, metabolic, and genetic pathways (95–97). Prior to the change in amplitude and frequency of the secretion of gonadotropin-releasing hormone (GnRH) from the hypothalamus that defines the initiation of ovine puberty, the hypothalamus of the pre-pubertal ewe is highly sensitive to negative feedback from estradiol. Estradiol, produced primarily from the pre-pubertal ovary, inhibits GnRH secretion and the release of follicle stimulating hormone (FSH) and luteinizing hormone (LH), without which ovulation does not occur. Phytoestrogens, such as equol, may exert a similar inhibitory effect in the ewe-lamb, particularly when it is present in combination with other factors that are known to delay puberty onset, such as poor nutrition, inadequate exposure to short-day photoperiod patterns, or a lack of social interaction (98).

Comparatively, in juvenile rodents, the effects of phytoestrogens on markers of puberty onset appear to be inconsistent, and depend on dosage (99, 100). At concentrations comparable with low level dietary exposure, genistein induces hyperplasia of mammary tissue in the pre-pubertal rat, but at concentrations comparable with high level dietary exposure reduce both alveolar development (99, 100) and the sensitivity of mammary tissue/gland to estradiol (101). In rodents, in cases where sexual maturity is advanced by phytoestrogen exposure, the estrous cycles are very irregular at the commencement of puberty (102–104). While the mechanism that leads to the oligomenorrhea is not known, several studies support the notion that pre-pubertal exposure to estrogen-like compounds, including genistein, alters hypothalamic kisspeptin expression and leads to a lower density of kisspeptin expressing neurons and fibers in the anteroventral periventricular and arcuate nuclei (102, 105). Kisspeptin has a well-established role in the timing of both puberty and the estrous cycle, playing a critical role in estrogen feedback to the hypothalamus. Because GnRH neurons do not express estrogen receptors, the mechanism of steroid feedback remained a mystery until the discovery of kisspeptin (106). The discovery that kisspeptin neurons do express estrogen receptors, and that substances released from kisspeptin neurons (including kisspeptin, neurokinin B, and dynorphin) can stimulate GnRH neurons, provided a mechanism for steroid feedback. Any lowering of the activity of the kisspeptin signaling pathway can compromise the normal steroid feedback to GnRH neurons (107–111). Theoretically, a decrease in the stimulation of GnRH neurons by kisspeptin could account for the delayed pubertal onset and impaired ovulation that is observed after neonatal phytoestrogen exposure (4, 102, 112–116). Investigation of the neuronal circuitry that leads to GnRH release in phytoestrogen-exposed ewe lambs may shed further light on not only whether puberty is physiologically delayed, but also whether there are more permanent effects on the hypothalamic-pituitary-gonadal axis.

In rats, phytoestrogen exposure during the fetal and early neonatal stage is associated with long term alterations to the estrous cycle after the exposed individuals reach puberty, with prolonged suppression of ovarian cycling (117) and inhibition of LH secretion (118). Neonatal exposure to genistein might create a more hostile uterine environment that is less capable of supporting embryo implantation (103, 119). In addition to these reproductive consequences, *in utero* exposure to estrogenic compounds also impairs immune function in rats (120), increases the incidence of cancerous lesions in the uterus (121, 122), and impairs the development of estrogen-sensitive organs, particular secondary sex organs (123, 124).

### Phytoestrogens Have Conflicting Effects on Reproductive Function in Adult Females

The effects of phytoestrogen exposure in the adult female remain unclear, with a spectrum of effects reported across species, ranging from beneficial to injurious. Adult mice exposed to estrogen-like compounds, for example, develop irregular estrous cycles, with more atretic follicles and an absence of *corpora lutea* (90). In women, dietary isoflavone intake is associated with an abnormally short luteal phase (125). More broadly across species, phytoestrogens have been linked to reproductive abnormalities, including an increase in uterine weight in cheetahs (126), reduced fertility in rhinoceros (127), and altered ova composition and estrous cyclicity in several bird species (112). In the context of clover disease, morphological changes in the follicles, along with reduced fertility, have been observed in mature ewes grazing estrogenic pasture, as outlined further below. Briefly, exposed ewes present excessive numbers of small to medium ovarian follicles with inadequate antrum formation, increased uterine fluid and subacute inflammation of the endometrium, and a reduction in the depth of cervical crypts and cervical squamous metaplasia (3, 6, 13, 128).

In contrast, in adult humans, isoflavone has been reported to have beneficial effects on fertility. In preconception cohort studies, higher intake of isoflavone was associated with improved fecundity and fertility (129, 130) and the re-instatement of ovulation in anovulatory cycles (131). In women with polycystic ovarian syndrome, supplementation with phytoestrogen extract of *Cimicifuga racimosa* increased plasma progesterone and endometrial thickness, suggesting that the phytoestrogens facilitated ovulation (132). It should be noted that the metabolites that are produced from phytoestrogens vary between ruminant and monogastric species, which may partly account for some of the species-specific outcomes of exposure to phytoestrogens. Several isoflavones, and their metabolites, have been suggested to have antioxidant effects and to mitigate inflammation (for an extensive review, see 133). These properties are likely responsible for the beneficial effects of isoflavones in cancer models (134) and in some cases, improvements in fertility. Reactive oxygen species (ROS) have a dichotomous role in reproduction, whereby particular levels of ROS are necessary for the processes of oocyte maturation, ovulation (135), and for spermatozoa to undergo capacitation and the acrosome reaction (136). Yet excessive levels of ROS are detrimental to gamete function and quality (136–138). In many cases, supplementation with antioxidants can improve gamete function and fertility *in vivo* (139, 140) and *in vitro* (141–144). Because phytoestrogens are known to reduce pro-inflammatory cytokines in experimental models of encephalitis (145, 146), any modulation of the immune response by phytoestrogens could either promote or inhibit normal reproductive function. In the female for example, non-specific inflammation can alter endometrial receptivity, impair tissue repair and remodeling, and affect trophoblast-endometrial interaction (147, 148).

#### A Meta-Analysis of the Effect of Phytoestrogen Exposure on Ovulation in Sexually Mature Females

It is clear that the impact of phytoestrogens on reproduction varies between studies, and that that variability is probably caused by differences in the dose of phytoestrogens and the timing of delivery. To tease apart the factors that determine the outcomes of phytoestrogen exposure, we performed a meta-analysis to clarify the impact of phytoestrogens on ovulation. Our logic was that ovulation is a key determinant of female reproductive performance. Major databases such as Pubmed, Cochrane Library, Google Scholar, and ResearchGate were searched using combinations of the following keywords: phytoestrogens, ovulation, ovary, sheep, clover, clover disease, isoflavones, coumestans, soy (i.e., not all terms were included in every search). The search included articles with at least an abstract in English. The end date of the database search was October 2020. Twenty-five studies were initially selected when the abstract indicated that the data would be relevant. Seven studies were selected for analysis after we applied the inclusion and exclusion criteria that are listed in **Table 1**, and the information and data extracted are listed in **Table 2**.

**Table 1:** Inclusion and exclusion criteria used in the meta-analysis.

**Inclusion criteria**
The phytoestrogen was a flavonoid
Ovulation was assessed by visualization of the corpus luteum/palpation (for mammals)
The route of administration was oral
**Exclusion criteria**
There was no control group
The ovulation was assessed only by an increase in plasma progesterone
Numerical outcome data were not provided
The phytoestrogens were administered by injection

**Table 2:** A summary of characteristics for studies included in the meta-analysis.

(First author, year)	Abbreviation	Treatment		Control		Detection of ovulation	Species
		Diet	Number of subjects	Diet	Number of subjects		
(Adams et al., 1979) (149)	ADAMS79B	Pasture of Yarloop subterranean clover for 3 years (isoflavones)	99	Non-estrogenic pasture	78	Laparoscopy	Sheep (Merino)
(Smith et al., 1979) (114)	SMITH79A	Lucerne pasture (coumestrol) for 2 months	80	Non-estrogenic grass pasture	80	Laparoscopy	Sheep (Perendale)
	SMITH79C	Pelleted lucerne (coumestrol) for 3 months	49	Pelleted non-estrogenic lucerne	49	Laparoscopy	Sheep (Perendale)
(Hashem and Sallam, 2012) (150)	HASHEM12	Berseem clover pasture for 50 days	6	Corn silage	6	Transrectal ultrasonography	Sheep (Barki X Awassi)
(Adams et al., 1981) (151)	ADAMS81	Yarloop subterranean clover pasture for 3 years (isoflavones)	49	Non-estrogenic pasture	53	Laparoscopy	Sheep (Merino)
(Santhosh et al., 2006) (152)	SANTHOSH06	*Rhaphidophora pertusa* (flavonoid) covering one estrous cycle	4	Rice gruel during entirety of estrous cycle	12	Rectal palpation	Dairy cows
(Bennetau-Pelissero et al., 2001) (153)	BENNETEAU01	Genistein-enriched diet for one year	19	Normal diet	19	Histology of ovary	Rainbow trout
(Li et al., 2014) (154)	RONG14	Genistein-enriched diet from weaning to week 7	6	Normal diet	6	Histology of ovary	Mice (C57BL/6J)

##### Statistical Analysis

The results of each study were considered as binary (ovulation or no ovulation), and the odds ratio was used as the measure of effect size. A random effect model, with the DerSimonian-Laird (DSL) method (155), was used to evaluate the effect of phytoestrogens on ovulation occurrence because the conditions differed between studies. R studio software (156) was used for this meta-analysis, with the packages *rmeta* and *meta* and the function *meta.DSL*. For odds ratios, computations were carried out on a log scale to maintain symmetry in the analysis. Visualization of the results was done with the functions *summary* and *plot*, which were used to confirm heterogeneity, the individual odds ratios, and confidence intervals. A Funnel plot was created to evaluate publication bias, with a test for funnel plot asymmetry, based on rank correlation or linear regression methods (function *metabias*).

##### Results

**Figure 2:**
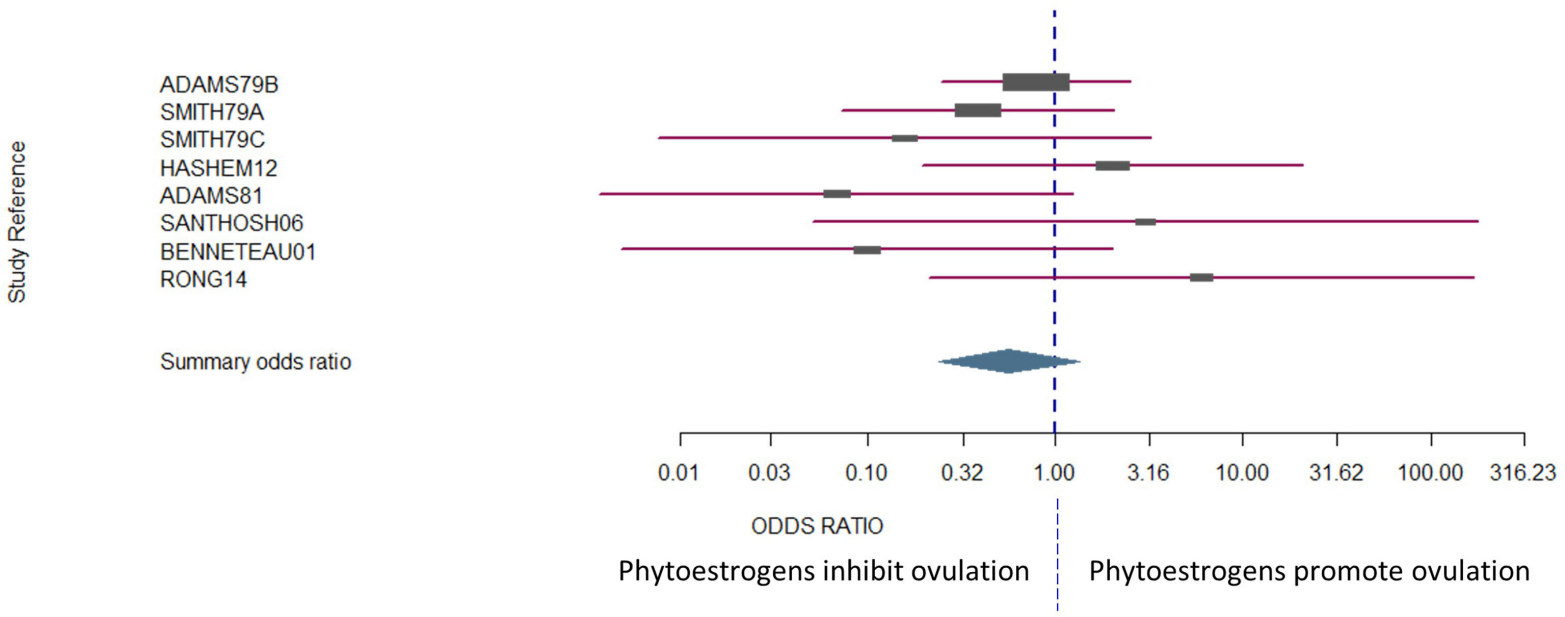
The confidence interval for each study is given by a horizontal line, and the point estimate is given by a square whose height is inversely proportional to the standard error of the estimate. The summary odds ratio is represented by a diamond with horizontal limits at the confidence limits and width inversely proportional to its standard error (R documentation). An odds ratio higher than 1 means that the treatment is more effective than the control and vice versa (whereby 1 means null effect). In the present analysis, the positioning of the summary odds ratio shows that phytoestrogen exposure in sexually mature females inhibits ovulation.

The number of animals in the studies ranged from 4 to 99. Laparoscopy was the most common method used to assess whether ovulation had occurred [one outcome per study, with the exceptionof Smith et al. (114)]. Four of the studies were conducted in sheep, three of which were conducted in Western Australia (7, 114, 149) and the fourth in Egypt (150). There was no bias across the included studies (P = 0.8349, **Figure 2**).

The results exhibited a large heterogeneity between studies (**Figure 2**), which can be primarily explained by differences in study design, including mainly species and sample size. However, the Woolf’s test for heterogeneity (X^2^) returned a p-value of 0.3157, and a between-studies variance of 0.23. Confidence intervals were notably wide, particularly for the studies that had fewer subjects in each experimental group (**Figure 2**).

##### Conclusions

It can be concluded that, depending on species, there remain contradictions around the impact of phytoestrogens on ovulation. It may be proposed that phytoestrogens inhibit ovulation in sheep, because that outcome seemed to emerge clearly from the data in the largest and the most statistically powerful of the studies. Interestingly, other phytoestrogens, non-flavonoids, have been reported to induce ovulation in women with polycystic ovarian syndrome or with anovulatory cycles (132). The impact of phytoestrogen on ovulation will be clarified only by further studies that are designed to control other external factors that can affect ovulation, such as metabolic status (157, 158).

### Male Reproductive Function

Estrogen was once considered a female hormone, but evidence over the past few decades has shown that it is critical for normal male reproductive development and function. In fact, estrogen appears to be essential for normal male reproductive function because mice with the ERα gene knocked out are sterile (159–161). Negative feedback from estrogen contributes to the activity of the hypothalamic-pituitary axis (162), and estrogen signaling plays a role in fluid reabsorption in the epididymis (163). Less is known about the effect of exogenous estrogenic compounds in the male than in the female, though there are several potential mechanisms for these agents to impact on the spermatogenic cycle. In primates and mice, ERβ is present in Leydig cells, Sertoli cells, and in the nuclei of epithelial and stromal cells throughout the reproductive tract, while ERα seems primarily to be present in the accessory sex glands and efferent ductules (164, 165). The disruption of ERα in male mice results in compromised dynamics of fluid resorption in the efferent ductules, an increase in the secretion of chloride ions into seminal fluid, an increase in abnormal sperm morphology, an inhibition of sodium transport in the testis and ultimately reduced fertility (159, 160, 163). Phytoestrogens that can bind to ER receptors would therefore be expected to alter testicular function, sperm production, and sperm quality.

In man, phytoestrogen exposure has a spectrum of effects (**Table 3**). Although the effects are generally more subtle than the marked changes in females (4, 120, 126), those effects again depend on timing, with exposure during sensitive developmental periods, particularly *in utero*, leading to compromised reproductive function. Maternal isoflavone intake has been linked to hypospadias in male infants (182, 183) and lower testicular steroidogenic activity in neonatal rats (178). Similarly, as for the female, in the male conflicting results have been reported for the effects of phytoestrogen exposure during adult life. Several studies have been reported no effect in humans (166, 170), rodents (184–186), and rabbits (187), while other studies in the same species have associated phytoestrogen exposure during adult life with decreased sperm production, lower blood testosterone concentration, and reduced testicular weight (167, 168, 173, 174, 188, 189). These conflicting results can probably be attributed to several differences between studies, including phytoestrogen dosage, route of administration, length of exposure and, in the case of human studies, demographic biases including variation in age, ethnicity, and systemic health. Moreover, none of the studies reported the concentration of phytoestrogens in the seminal plasma. Thus it is not clear to what degree these compounds can infiltrate the reproductive tract or whether other indirect pathways are involved.

**Table 3:** The effect of phytoestrogen exposure on male reproductive function across several species and routes of exposure.

Species	Phyto-estrogen or source	Route	Period of exposure	Effects on sperm production and quality	Other changes to reproductive capacity	Effects on endocrinology	Reference
Human	Soy	Dietary	Adult	No effect	No effect	No effect	(166)
				Decreased concentration	No effect	N/A	(167)
				No effect	No effect	Decreased testosterone	(168)
	Daidzein, genistein	Dietary	Adult	Low motile sperm count	N/A	N/A	(169)
				Decreased concentration and motility, increased abnormalities	N/A	Increased infertility	(10)
		Oral	Adult	No effect	No effect	No effect	(170)
		Intraperitoneal injection of mother	Fetal	Decreased concentration	Low epididymal density and quality, deteriorated testicular architecture, reduced total pups sired	Decreased testosterone	(171)
	Genistein	Dietary	Birth to adulthood	Reduced cauda epididymis sperm reserve	Penis underdevelopment, lower epididymal weight	Decreased testosterone	(9)
			Neonate, adult	N/A	Abnormal testis, increased inflammation of testis, increased rates of infertility.	N/A	(172)
			Conception to adulthood	Decreased concentration (epididymal)	Reduced haploid germ cells in testis, decreased size of seminal vesicle	N/A	(173)
	Soy	Dietary	Adult	Increased abnormalities in sperm morphology	N/A	N/A	(174)
	Thai Mucuna seed (isoflavone)	Dietary	Adult	Increased sperm concentration	N/A	N/A	(175)
Rodent	Pueraria mirifica (isoflavone)	Dietary	Adult	N/A	Lipid peroxidation of epididymal sperm was significantly increased	Disrupted steroid regulation of epidydimis, significantly reduced fecundity	(176)
	Genistein, daidzein, glycitein	Oral	Neonate	No change	No change	No change	(103)
		Dietary	Adult	Decrease in the weights of the left testicle, seminal vesicle, sperm count	Decreased sperm motility	Decreased testosterone hormone, no change in plasma estradiol	(177)
	Lignans	Dietary	Adult	Increased sperm concentration	N/A	Leydig cell number increase	
	Isoflavones (soy)	Dietary	Fetal	Increased proliferation of Leydig cells	N/A	Reduced steroidogenesis in adulthood	(178)
		Dietary	Fetal	No effect on gametogenesis	N/A	No impact on testosterone	(179)
Rabbit	Soy, lignans	Dietary	Adult	Decreased sperm concentration	N/A	Decreased libido, testosterone, seminal plasma fructose. No effects on number of offspring	(180)
Bat	Coumestrol	Dietary	Adult	N/A	N/A	Increased testicular weight, loss of typical histological structure of testis	(181)

N/A is specified in cases where the parameter was not measured in that study.

While the impact of phytoestrogen exposure on male reproduction is poorly understood, *in vitro* work suggests several isoflavones and flavonoids can influence the functionality of spermatozoa. The exposure of ram spermatozoa to physiological concentrations of equol *in vitro* decreases sperm motility, increases ROS, increases membrane fluidity, and increases DNA fragmentation (11). Genistein (8, 190, 191) and myricetin (192) have been reported to cause premature capacitation and acrosome loss in human, boar, and mouse spermatozoa, and inhibit the acrosome reaction in bull spermatozoa. In contrast, other studies have reported that the inclusion of isoflavones *in vitro* improves sperm viability and function (193–195). One possible confounding factor is that several isoflavones, including the widely tested genistein, can act as an antioxidant and mitigate oxidative stress (196, 197), thus improving sperm function. It is possible that the benefits of the antioxidant action can, at certain concentrations, mitigate any negative impacts from the estrogen-like actions of phytoestrogens.

## Clover Disease: Have We Overlooked a Detrimental Impact on the Interaction Between Spermatozoa and the Female Reproductive Tract?

Despite its categorization as a female oriented issue, mounting evidence suggests that the subfertility and infertility that is associated with clover disease is likely far more complex. Since a 1960’s report that wethers on estrogenic pasture exhibited mammary development, lactation, and pathology of the bulbourethral glands (198), there is little investigation into the impact of phytoestrogens on ram reproduction. Similarly, there is no information on how ram spermatozoa perform in the female tract after phytoestrogen exposure in either, or both, sexes. The profound effects of phytoestrogens on male fertility in other species (**Table 3**) suggests strongly that phytoestrogen impact on ram reproductive function may be a critically overlooked component of clover disease. Of note, human males exposed to estrogenic compounds at relatively high dosages or duration have smaller seminiferous tubules and impaired spermatogenesis (199). Roosters and rats exposed to estrogenic compounds have lower testosterone production (200, 201), more oxidative stress, and reduced sperm function (5). In the boar, estrogenic compounds cause premature capacitation (202) and a stronger immune response to spermatozoa in the female tract (203).

We propose that exposure to phytoestrogens could compromise both the male and the female, as well as interactions between spermatozoa and the female tract, creating conditions within the female reproductive system that are hostile, more so than usual, to spermatozoa. The exposure to exogenous estrogens could drastically impair the ability of spermatozoa to traverse the female tract and successfully fertilize, which could partially account for the poor fertility in sheep that graze estrogenic pasture (4, 204). The addition of physiological concentrations of equol to ram spermatozoa *in vitro* negatively alters sperm function, with decreased sperm motility, increased ROS, increased membrane fluidity, and greater DNA fragmentation compared to non-exposed ram spermatozoa (11).

One obvious factor that will influence the interaction between spermatozoa and the ewe reproductive tract is the morphological changes to the female reproductive tract that are observed after phytoestrogen exposure (3, 6, 128). Prolonged grazing of estrogenic pasture has been found to induce a loss of crypts and folds in the cervix of ewes (4, 6, 13). The crypts of the ovine cervix represent “privileged pathways”, potential routes where conditions of cervical mucous flow, composition, and viscosity are conducive to the successful progression of spermatozoa toward the oocyte (205, 206). Thus, the morphological changes that are observed in female tract and in the sperm physiology after exposure to phytoestrogens could synergize to compromise the ability of spermatozoa to traverse the female reproductive tract.

The ability of spermatozoa to navigate the female reproductive tract is not merely a function of motility, but rather involves dynamic interactions between the male gamete and the tract environment. Particularly noteworthy in the context of reproduction after animals are exposed estrogenic compounds, is the role of ovarian steroids in the production and composition of cervical mucous, and in the immune response that is stimulated by the presence of the incoming spermatozoa (205, 207). Exogenous hormones, such as those that are used to synchronize the estrous cycle of ewes, are known to vastly alter the composition of cervical mucous thereby impairing the function of spermatozoa, leading to a reduction in fertility (205, 207–210). Infertility in ewes that graze sub clover species that are estrogenic could be partially due to changes in cervical mucous that are induced by phytoestrogens. During the follicular phase, the viscosity of the cervical mucous decreases in response to rising estrogen concentrations, lowering the barrier to sperm migration through the female reproductive tract. That change optimizes the conditions in preparation for ovulation and potential insemination (205, 207). Very early studies in sheep showed that the viscosity of mucus was lower after chronic exposure to estradiol and in ewes with clover disease (12, 211). However, the lower mucus viscosity in ewes with clover disease was also correlated with reduced fertility (212). This suggests that a factor other than viscosity, such as the biochemical composition of mucus following long term phytoestrogen exposure, is less favorable to sperm transport.

While exogenous estrogen is known to alter the female immune response and the composition of seminal plasma in the male (163), it is not known if phytoestrogens elicit similar effects. When seminal plasma enters the female tract immune cells are recruited and activated, releasing cytokines and chemokines that play a role in the sperm selection process in the female tract (213). In mammals, the magnitude of this immune response varies in response to endogenous estrogen (203), exogenous hormones (214), and specifically in sheep, between breeds (205). There are reports of the production of anti-sperm antibodies in the female reproductive tract after insemination, though a predominant focus of immune studies has been the known immunogenic properties of seminal plasma (215, 216). Because some of the constituents of seminal plasma have an immunomodulatory effect (215, 217), anything that influences the composition of the seminal plasma could affect the female immune response, with an impact on sperm survival. Greater numbers of leukocytes in cervical mucus increases sperm capacitation and the acrosome reaction (218). Because leukocytes produce ROS at far higher levels than do spermatozoa (219), the production of ROS might be the underlying mechanism for the promotion of these events. It will be worthwhile to investigate whether phytoestrogens alter the composition of seminal plasma, and if that then modulates the immune response that is mounted by the female after copulation.

In summary, the combination of loss of privileged pathways for sperm in the cervix, changes in the composition of cervical mucous, and changes in the immune response of the female to spermatozoa all represent possible mechanisms that could contribute to the fertility issues that occur in sheep when they graze sub clover (**Figure 1**). Investigation is warranted into male-female gamete interactions after either sex is exposed to phytoestrogens.

## Conclusions and Recommendations for Future Management of Ovine Clover Disease

There are two clear rationale for further research into clover disease; there are clear agricultural benefits to be gained, and this syndrome in sheep presents a unique opportunity to develop a model of the reproductive consequences of phytoestrogen exposure, particularly during critical developmental periods. The review of the known literature points logically to several potential sites of action, and mechanisms of action, of phytoestrogens on sheep reproduction. This includes estrogen receptor binding, genomic and epigenetic alterations and immune modulation, at sites including the reproductive tract, brain and gametes of both the male and female. It is likely that a systemic interplay between those factors is responsible for the spectrum of pathological changes that are observed in ovine clover disease, though that itself requires clarification. In our view, it is necessary not only to understand the consequences of exposure to phytoestrogens on gamete production in both sexes, but also to understand how that exposure alters the interaction of the gametes in the female reproductive tract. An interesting avenue of research would be to explore the physiological and genetic basis of individual susceptibility to phytoestrogens. The identification and strategic breeding of resilient animals is a logical start to address the issue, as well as the removal from breeding of persistently sub-fertile individuals and the early identification of susceptible flocks. Those strategies could be used in parallel with pasture renovation to eliminate estrogenic clover.

## Author Contributions

KP drafted, edited and wrote the final manuscript. FC conducted the meta-analysis. DB and JS reviewed, edited and contributed to the manuscript. All authors approved the final version of the manuscript.

## Funding

KP is supported by the Lefroy Bequest, and DB was supported by a Meat and Livestock Australia grant (P.PSH.1138).

## Conflict of Interest

The authors declare that the research was conducted in the absence of any commercial or financial relationships that could be construed as a potential conflict of interest.

## Publisher’s Note

All claims expressed in this article are solely those of the authors and do not necessarily represent those of their affiliated organizations, or those of the publisher, the editors and the reviewers. Any product that may be evaluated in this article, or claim that may be made by its manufacturer, is not guaranteed or endorsed by the publisher.
